# “Price-Quakes” Shaking the World's Stock Exchanges

**DOI:** 10.1371/journal.pone.0026472

**Published:** 2011-11-02

**Authors:** Jørgen Vitting Andersen, Andrzej Nowak, Giulia Rotundo, Lael Parrott, Sebastian Martinez

**Affiliations:** 1 CNRS, Institut Non Linéaire de Nice, Sophia Antipolis, Valbonne, France; 2 Department of Psychology, Warsaw University, Warsaw, Poland; 3 Department of Economics, University of Tuscia, Viterbo, Italy; 4 Complex Systems Laboratory, Départment de géographie, Université de Montréal, C.P. 6128 succursale “centre-ville” Montreal, Québec, Canada; 5 CRA 1 No. 18A-12 Department of Mathematics, Universidad de los Andes, Bogota, Colombia; Tel Aviv University, Israel

## Abstract

**Background:**

Systemic risk has received much more awareness after the excessive risk taking by major financial instituations pushed the world's financial system into what many considered a state of near systemic failure in 2008. The IMF for example in its yearly 2009 Global Financial Stability Report acknowledged the lack of proper tools and research on the topic. Understanding how disruptions can propagate across financial markets is therefore of utmost importance.

**Methodology/Principal Findings:**

Here, we use empirical data to show that the world's markets have a non-linear threshold response to events, consistent with the hypothesis that traders exhibit change blindness. Change blindness is the tendency of humans to ignore small changes and to react disproportionately to large events. As we show, this may be responsible for generating cascading events—pricequakes—in the world's markets. We propose a network model of the world's stock exchanges that predicts how an individual stock exchange should be priced in terms of the performance of the global market of exchanges, but with change blindness included in the pricing. The model has a direct correspondence to models of earth tectonic plate movements developed in physics to describe the slip-stick movement of blocks linked via spring forces.

**Conclusions/Significance:**

We have shown how the price dynamics of the world's stock exchanges follows a dynamics of build-up and release of stress, similar to earthquakes. The nonlinear response allows us to classify price movements of a given stock index as either being generated internally, due to specific economic news for the country in question, or externally, by the ensemble of the world's stock exchanges reacting together like a complex system. The model may provide new insight into the origins and thereby also prevent systemic risks in the global financial network.

## Introduction

Like earthquakes, financial crises appear to be ever recurrent phenomena with the unfolding of a given crisis strongly dependent on the history that led up to the crunch. Whereas the continuous build up of stress from tectonic plate movements is well understood to be at the origin of earthquakes, the causes behind financial distress remain unclear, with explanations often sought in singular events.

Systemic risk in general refers to the risk of collapse of an entire system. In a financial context, systemic risk can for example ocour when the financial distress of major banks spreads through ripple effects to other banks who have acted as counterparties in common transactions with the banks in trouble [1]. The traditional litterature on financial systemic risk usually deals with bank contagion, looking at exposures where the default by one bank would render other banks insolvent. Other more recent studies however take a larger look at the financial system by studying contagion in financial markets but typically only for one market at a time [2]–[6]. A general survey of the topic can be found in [2]. In [3], [4] measures of systemic risk were proposed indirectly via econometric techniques such as principal components analysis and Granger-causality tests of four sectors: hedge funds, banks, brokers and insurance companies. It was found that the level of systemic risk has increased over the past decade because the four sectors have become highly interrelated. In [5], [6] it was similarly reported that the US S&P500 market has beome prone to systemic collapses since 2002 but using a different technique, the index cohesive force (ICF), which measures the balance between stock correlations and partical correlations (defined via an subtraction of the index). The study in [5], [6] provides a new way of looking at market dynamical states and stability and transitions between such states, similar to the motivation of the study proposed here. Crucial to such efforts is to understand how disruptions can propagate througout the system, as well as understand how correlations change as the states of the markets change [7]. For example in [3], [4] it was shown how correlations increase during market crashes. For other studies on market correlation structure see also [8], [9] and [10], [11]. Very few studies have been made of systemic risk at the largest possible scale, the world. See however [12]–[14] showing some very interesting studies including the worlds financial markets. We argue that a proper understanding of systemic risks necessitates an understanding at the system level of how disruptions can be created and propagate across financial markets. We therefore suggest a top-down view on global risks, but stress the relevance of individual countries, by looking at the world's network of stock exchanges instead of focusing on individual markets. Of particular interest will be to suggest new approaches to the risk of contagion where the transmission of financial shocks can propagate across countries. It is in this context that we present a model that takes a holistic view of how pricing takes place in the world's stock markets.

Using empirical data we first illustrate how, in particular, big price movements in a given stock exchange have impacts on the other stock exchanges world-wide, whereas small price movements have no impact on the other exchanges and go unnoticed. Such a dynamics resemble very much the dynamics behind earthquakes where small stresses first build up locally without any propagation. However once sufficiently large, a stress will eventually be released and propagate in sudden bursts. We use this insight to introduce a network model of the world's financial system. In the model each stock exchange is represented as a block in a network that links any two blocks with a spring of variable strength. As will be explained below, the price movements of the stock exchanges are then partially created by the stick-slip motion of the network of blocks, something very similar to ideas originally introduced by Burridge and Knopoff (BK) [15] to describe earthquakes caused by tectonic plate movement. This allows a direct study of memory effects in the global financial system, with stresses that build up over time and are released in sudden bursts much like what is seen during seismic activities of earthquakes. Thus, we emphasize a description where the price movements of any given stock market can not be solely understood by looking at the level of the individual stock exchange and propose that a proper characterization needs to account for system-wide movements at the global level.

Our objective is to study how stresses in the global financial system of stock exchanges build up and propagate. In our model, stress enters the system because of price movements of the indices, represented by displacements of the blocks. Stress can either be locally generated due to economic news for a specific index, or globally generated due to the transfer of stress when a large price movement happens for a given stock index. The idea is that a large (eventually cumulative) price movement of a given stock index can induce stresses on other stock indices world-wide to follow its price movement. Similar to the BK model of earthquakes, we assume a “stick-slip” motion of the indices so that only a large (eventually cumulative) movement of a given index has a direct impact in the pricing of the remaining indices world-wide. In this line of thinking “price-quakes” can happen in the global financial system as cascades of big price movements originate from one corner of the globe and propagate world-wide like falling bricks of dominos. We are thus representing the global financial system as a complex system, characterized by important memory effects and path dependence.

A key principle in finance states that as new information is revealed, it immediately becomes reflected in the price of an asset and thereby loses its relevance [16], [17]. We suggest to combine this principle with a behavioral trait which reflects the tendency of humans to reply in a nonlinear fashion to changes, placing emphasis on events with big changes and disregarding events with modest information content. This is in agreement with experiments made in psychology which have shown that humans react disproportionally to big changes, a phenomenon called change blindness since only large changes are taken into account whereas small changes go unnoticed [18]–[20].

Change blindness has been reported in laboratory experiments even when the participants are actively looking for changes. When small rapid changes occur to photographs observers often miss these changes, provided that the change is made during a saccade [21], a flashed blank screeen [22], a blink [19], or some other visual disruption [23]. For a review article on change-blindness see e.g. [24].

As new information is produced at a given exchange, say the opening or closing price of that particular market, it becomes part of the information that other exchanges may or may not use in their pricing. With the existence of futures contracts, this information, as well as other economic news, is in principle priced in instantaneously, even outside the opening hours of exchanges. However if one uses the amount of trading volume as a proxy for the relevance of the reaction to new information, it is the opening (or respective closing) price that determines the most important moment where new information generated prior to the current exchange's trading session becomes priced in. Thus, in the following we will use the opening/closing (open/close) prices, which usually correspond to times when the trading volume is highest, as the values of the stocks/indices that become priced in with new information.

## Analysis

Imagine a trader who at the opening of the Tokyo stock exchange tries to price in new world-wide information coming from the other stock exchanges about what happened since the markets last closed in Tokyo. We conceive that she/he does so by taking into account both the release of local economic news in Japan (that happened since the previous day's close) as well as by seeking out news about how other markets performed **after** the markets closed in Tokyo. Because of the time zone differences, new information at the opening in Tokyo would include the price difference between the open and the close the day before for the European and American markets. For the Australian market, however, this would include the price difference between the close of the day before and the open the same day, since this market is the first market to open world-wide, and opens before the Japanese markets. We postulate a universal behavioral mechanism in the pricing done by traders evaluating two different terms i) local economic news ii) *big cumulative* changes from other stock exchanges weighted by their importance (in terms of capitalization) and their relatedness (in terms of geographical positioning representing e.g., overlap of common economic affairs or importance as trading partners).

At time 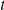, the trader of a given stock exchange 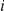 estimates the price 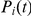 of the index as 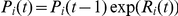, with 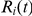 the return of stock exchange 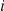 between time 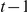 and 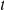:
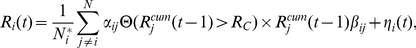
(1)

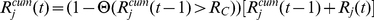
(2)

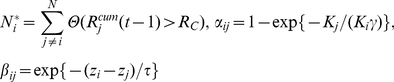
(3)

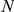
 is the total number of stock exchanges. 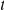 stands for the time of the close (respective open) of exchange 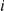 whereas 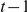 is the time of the last known information (close or open) of exchange 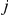 known at time 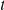.

The 5 parameters of the model are: 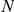, 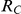 - the threshold return, 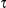 - the time scale of the impact across time zones, 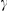 the scale of impact from capitalisation and 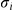 the standard deviation in the noise term 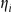.

The reasoning behind (1)–(3) goes as follows: at the opening/close of a given stock exchange 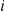 new internal economic news is priced in via the second term in (1), 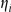, which represents internal economic news only relevant for the specific index 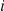. The first term in (1) describes the fact that traders look up what has happened in other stock markets world-wide, but it is only when a sufficiently large (eventually cumlative) price move happens in another stock exchange 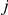 that it has an influence on the stock exchange 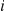. The use of the Heaviside 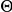-function ensures that the first term in (1) is zero for small cumulative moves of stock exchange 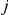, i.e. in this case stock exchange 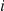 does not feel any influence from stock exchange 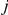. The pricing of stock exchange 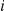 however receives the contribution 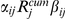 when a sufficiently large (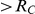) cumulative move happens at the stock exchange 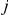. The two coefficients 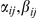 (explained further below) describe how big an influence a price move of stock index 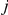 can have on the given stock index 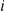. It is important to note that 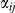 is assymetric, 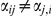 since the impact that a big price movement of stock index 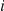 has on another stock index 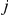 is *not* the same as the impact of the same big price movement of the stock index 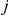 on the stock index 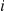. The factor 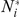 in (1) means that the index 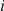 takes into account an average impact among the indices 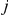 that have the condition in the Heaviside function fullfilled. (2) takes account of the fact that when a big (eventually cumulative) price move of stock exchange 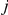 has had an impact on stock exchange 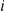 it becomes “priced in”. The “stress” due to the large cumulative move of stock exchange 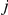, 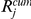, is therefore released and set equal to zero, something which is accounted for via the 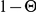 term in (2).


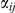
 is a coefficient that describes the influence of stock index 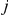 on stock index 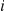 in terms of relative value of the capitalization 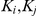 of the two indices. A large 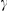 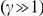 corresponds to a network of the world's indices with dominance of the index with the largest capitalization 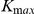. Presently this is the U.S. financial market, so choosing 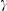 large corresponds to the case where pricing in any country only takes into account the movements of the U.S. markets as external information. On the contrary a small 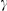 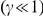 corresponds to a network of indices with equal strengths since 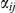 then becomes independent of 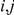. In addition we assume that countries which are geographically close also have larger interdependence economically, as described by the coefficient 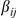, with 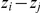 the time zone difference of countries 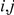. 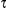 gives the scale over which this interdependence declines. Small 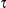 (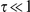) then corresponds to a world where only indices in the same time zone are relevant for the pricing, whereas large 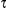 (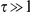) describes a global influence in the pricing independent of the difference in time zone. The structure of (1) is similar to the Capital Asset Pricing Model [25]–[27] since it predicts how an individual stock exchange should be priced in terms of the performance of the global market of exchanges, but with human behavioral characteristics included in the pricing.

Each index is composed of a given number of stocks. As such each index, or block, can itself be thought of as a spring-block system, where now each block in the sub-spring-block system is representing a given stock. This opens the possibility for a hierarchical description of the world's stock exchanges where the distress of a given stock can either influence directly another stock in another index, or indirectly through its influence on its index, can influence other indicies and thereby stocks world-wide. In the present study we will first concentrate on the direct interrelations between indicies, leaving out impacts from individual stocks to a future study [28].

The processing of news is a key building block of the model. It is however not alway that news will influence one stock index directly, but can instead influence single stocks, sectors or groups of stocks, possibly in different markets at the same time. Therefore the model may be viewed as a kind of factor model, since idiosyncratic shocks might have almost no effect on the index, as they might to a certain extent average out. Other factors (interest rates, oil prices, labor markets) with an impact on many stocks may have a noticeable effect on the index, possibly on all indices. As a result a large movement of an index is likely to stem from the impact of an important factor which is then also likely to have impact on stocks in other markets. Therefore the model can be thought of as simply filtering for large index movements, which of course may happen jointly in many markets, because they are caused by the same factor. (This paragraph was added using the remarks of one of the two anonymous referees).

It should be noted that memory effects are present in the model since it is the cumulative “stress” that determines when a block “slips”. In Self Organized Critical (SOC) systems, memory is known to be an essential ingredient for the criticality of the system [29]. Formally (1)–(3) describes a 2D BK model of earth tectonic plate motion [30], [31]. Our model can thus be seen as an extension of the 2D Olami-Feder-Christensen (OFC) model [30] where each block is connected to all other blocks with 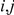-dependent coupling constants 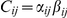. However, in the OFC model each block is only connected to its 4 neighbors and has only three (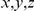-dependent) coupling constants. In addition, in our model, “out of plane” stresses are randomly (in both sign and magnitude) introduced via 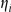 at each block instead of the constant (same sign) pull of the OFC model. (1)–(3) gives therefore an interesting perspective of looking at the world's financial system as a complex system with self-organizing dynamics and possibly similar avalanche dynamics as can be observed for earthquakes. Yet another interpretation of (1)–(3) is to view the world's financial system as set of coupled oscillators. The oscillations happen because of (2) where stresses are gradually built up and then released. Each stock exchange can therefore be seen as oscillating with a given frequency, and this oscillation can in turn influence the frequency of the other oscillators in the system leading to synchronization effects.

## Results

To verify the hypothesis that large movements in the stock exchanges play a special role and tend to lead to clustering of large movements, we have used empirical data to calculate the conditional probability that a given stock market's daily return, 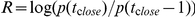 has the same sign as the daily return of the world market of indices. The data was downloaded from the website finance.yahoo.com and used the opening and closing price of the following 24 stock exchanges from 1/1/2000 to 1/10/2008: AORD (Australia), N225 (Japan), KS11 (South Korea) SSEC (China), HSI (Hong Kong), TWII (Taiwan), STI (Singapore), KLSE (Malysia), JKSE (Indonesia), BSESN (India), TA100 (Israel), CCSI (Egypt), FTSE (U.K.), FCHI (France), GDAX (Germany), SSMI (Switzerland), MIBTEL (Italy), AEX (Netherlands), ATX (Austria), MERV (Argentine), BVSP (Brazil), GSPC (U.S.), GSPTSE (Canada) and MXX (Mexico). Only days for which all stock markets were open were used in the analysis (each market has its own holidays).

We defined the index of the world market as:
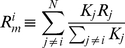
(4)with 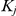 the capitalization of the 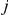
'th country's index. We use this index as a measure of the collective response of the world markets on a given day on the index 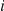. Notice the 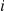 dependency since each index will have a specific impact from the other indicies world-wide. Notice also that since we want to avoid “self-impact” 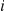 is excluded in the sum.

**Figure 1:**
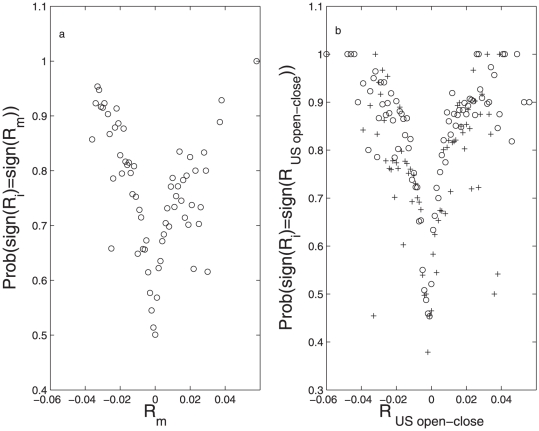
Illustration of change blindness: a large world market return (fig a) or US market return (fig b) impacts a given stock exchange, whereas small returns have random impact. a) Conditional probability that the daily return 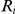 of a given country's stock market index has the same sign as the world market return defined by 
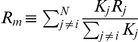
 with 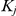 the capitalization of the 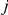
'th country's index. b) Conditional probability that the close-open (+: European markets; circles: Asian markets) return 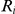 of a given country's stock market index following an U.S. open-close, has the same sign as the U.S. open-close return.

From Fig. 1a it is clear that when the world-wide index only exhibits small changes, little coherence is seen between the different country's movements. However, there appears to be a threshold after which large movements in the world-wide index lead to synchronization of the individual country's exchanges, with the majority tending to move in the same direction. Similar results have been found for individual stocks of a given stock market [10], [11]. This reinforces our claim that the stock markets world-wide should be considered as *one* system with *large* events playing a special role.

We then checked the specific assumptions in (1)–(3) that large movements of large capital indices should have a particular impact on smaller capital indices by looking at the effect of both the world market return and the US market return on the movement of individual stock exchanges (figure 1). Using the open-close return of the U.S. stock market gives a clear case to check for such a “large-move” impact. Since the Asian markets close before the opening of the U.S. markets, they should only be able to price in this information at their opening the following day. That is, the “open-close” of the Asian markets follow *after* a “close-open” of the US market with *no* stock market information in between. An eventual “large-move” U.S. open-close should therefore have a clear impact on the following close-open of the Asian markets. On the contrary, the European markets are still open when the U.S. market opens up in the morning, so the European markets have access to part of the history of the open-close of the U.S. markets. An eventual “large-move” U.S. open-close would therefore still be expected to have an impact on the following close-open of the European markets, but less so than for the Asian markets since part of the U.S. move would already be priced in when the European markets closed. Since the opening of the Asian markets by itself could influence the opening of the European markets, this furthermore could distort the impact coming from the U.S. markets. As expected, this effect is seen more clearly for the Asian markets compared to the European markets in figure 1b.

**Figure 2:**
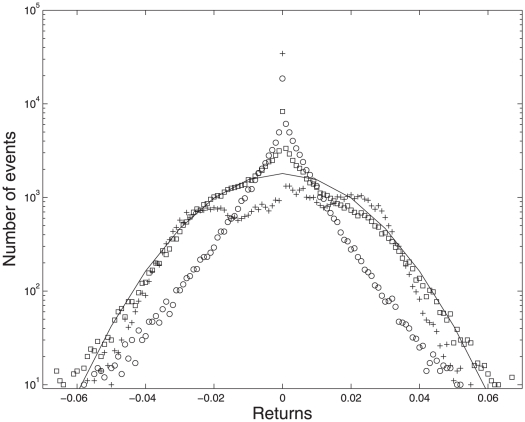
Impact of change blindness on market prices. Circles - observed returns 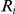. +'s - the term arising due to change blindness, 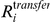. Squares - shows the difference 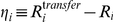 which according to (1)–(3) should be Gaussian distributed. Solid line represents a Gaussian distribution.

As an additional check on our assumption (1)–(3) we have constructed the difference 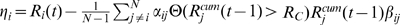 from the empirical data of 24 of the world's leading stock exchanges using daily data since the year 2000. According to (1) this difference should be distributed according to a Gaussian distribution. Using maximum likelihood analysis as given in the appendix we found the optimal parameters to be: (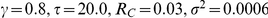). Figure 2 shows that for these parameter choices, our definition of price movements due to external (random) news does indeed fit a normal distribution. The obtained values of the optimal parameters suggest a fairly “global” network of stock exchanges with a large influence of pricing across time zones and pricing not only dominated by the largest capital index. A priori this seems in agreement with expectations. The value of 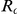 is furthermore consistent with the estimate one can retrieve independently by visual inspection of figure 1. Varying the parameters we estimate their significance to be within a factor of 2 of the values given. Lastly, given these optimal parameters, we predicted the sign of the open/close for each stock exchange using the sign of
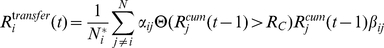
(5)

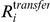
 describes the part of the return of a given stock index 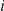 that is attributed due to large movements of other stock indices. Using in total 58244 events we found a very convincing 63% success rate of predicting the sign of the return of the open/close of a given stock exchange *ex ante*.

**Figure 3:**
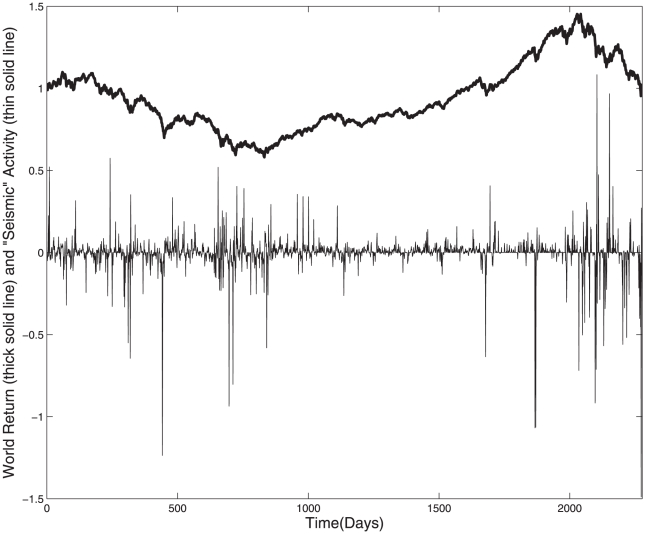
Seismographic activity (thin solid line) of price-quakes 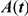
 **.** Thick solid line represents the world return index normalised according to capitalisation of the different stock indices.

**Figure 4:**
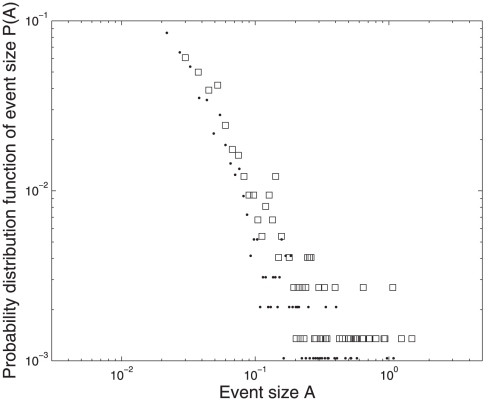
Inset to **figure 3**: Probability of the size 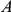
 **of a given seismic event.** Squares: negative returns, dots: positive returns.

In analogy with earthquakes, we now introduce a measure to determine the strength or “seismic” activity in the world's network of stock exchanges. To do so we suggest to consider each stock exchange as a seismograph which at any moment in time can measure the amplitude of the “wave of stress” imposed on it by the large price movements of the other stock exchanges world-wide. This quantity is given by 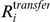 in (5). The global “seismic” activity at any moment of the world's stock exchanges can then be determined as an average of the measurement of each of the seismographs world-wide as: 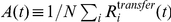 Using 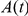 defined in this way one can investigate whether such activity could be used to characterize special periods with high “tremor” activity of the world's stock exchanges. Figure 3 shows the recordings of the “tremor” activity in the world's stock exchanges which resembles the recordings seen from seismographs of earth tectonic plate movements. The large event tail of the probability distribution function of the activity seen in Figure 4 shows the familiar power law behavior as seen in the seismic activity of earthquakes.

As mentioned in [29] memory effects at the system level in SOC systems are generated dynamically. Only when a SOC system has entered a steady state does the system exhibit long ranged correlations with power law events. In this sense the large event tail of the probability distribution function signals the presence of memory effects and a steady state of the global network of stock exchanges.

Most notable is a striking tendency for large “tremor” activity during down periods of the market. That is, the collective response of the network of stock exchanges world-wide seems to be stronger with larger “price-quakes” (positive as well as negative) when the world is in the “bear” market phase as compared to the “bull market” phase.

## Discussion

We have introduced a new model of pricing for the world's stock exchanges that uses ideas from finance [32], physics and psychology. The model is an extended version of the Burridge-Knopoff model that originally was introduced to describe earth tectonic plate movement. We have used an analogy with earthquakes to get a new understanding of the build up and release of stress in the world's network of stock exchanges and have introduced a measure that correctly captures the enhanced activity of price movements seen especially during bear markets.

In this sense our “seismic activity” measure gives yet another measure to assess phases of systemic risk much like the principal components analysis measure of [3], [4] and the index cohesive force of [5], [6]. However it would also be interesting to use our model for “tipping point” analysis using scenario analysis determining particularly dangerous moments of contagion in the financial system. Nonlinearity entered the model as the behavioral tendency of humans to react disproportionately to big changes. As predicted, such a nonlinear response was observed in the impact of pricing from one country to another. The nonlinear response allows a classification of price movements of a given stock index as either exogenously generated due to specific economic news for the country in question, or endogenously created by the ensemble of the world's stock exchanges reacting like a complex system. The approach could shed new light on risks on systemic failure when large financial price-quakes propagate world- wide [28].
